# The Midwinter National Meetings

**Published:** 1904-01-15

**Authors:** 


### DENTAL REGISTER.

Vol. LVIII. JANUARY 15, 1904. No. 1.

### THE MIDWINTER NATIONAL MEETINGS.

The midwinter meeting of the Institute of Pedagogics, the called meeting of the National Association of Dental Faculties, and the American Society of Orthodontists all convened during the week beginning Monday, December 28th, and ending Saturday, January 2d, at the Iroquois Hotel in Buffalo, N. Y. The meetings combined to draw the largest attendance ever held by any of these bodies, and there was so much doing all the time that everybody was kept busy and each to his liking. In addition a meeting of the Committee on the Fourth International Dental Congress held a three days' session and did a lot of necessary business in preparation for that important event. It is reported that all differences as to organization have been or will be soon adjusted and a harmonious meeting will be the result. A large sum of money, about thirty thousand dollars, will be needed to defray the expenses of the meeting, and a committee is busily engaged securing contributions from all local, district and national associations. Private members will pay a fee of ten dollars which will secure a copy of the proceedings as well. In these various ways it is expected that the desired funds will be forthcoming. While the time is short for the organization of so great a meeting it is confidently expected that every interest will be fully represented.

The meeting of the National Association of Faculties was called to hear a report of the committee appointed to devise a method estimating the value of credits for admission and advanced standing as well as for class grading by the plan known as the "*count system.*" A "*count*" means that a study has been pursued for one term or semester with four or five class recitations or twice as many laboratory hours each week. A certain number of counts are necessary for admission to college and a certain number are necessary before graduation. The system has been in use in many colleges and universities for a long time, but seems to be coming into favor in educational schools generally as it simplifies keeping records and giving advanced credits for work done in other schools. After a long discussion the matter was referred back to the committee for further preparation and report to the annual meeting in August. The other matter was the consideration of a resolution introduced at the last meeting, rescinding the law making a four-year course and a seven-month's term compulsory on all schools holding membership in the Faculties Association. The question was discussed during two sessions and then laid on the table until the August meeting, as the President ruled that action could not be taken legally at this meeting. Because of the fact that a number of those present came long distances to attend this meeting, and with instructions from their faculties as to how to vote, it was decided that a general discussion of the propriety of the resolution should be had at this time, so that a concensus of opinion at least might be secured. In the discussion which followed it was apparent that about half of the schools wanted to go on with the four-year course, while from twelve to fifteen wanted an eight or nine months, session with a high-school graduation for admission and a three-year course. As nearly as could be determined, the balance wanted to return to a three-year course with the present admission standard and a six or seven months' term. It seems impossible to reconcile all these ideas and come to any conclusion that all would accept, and it was evidently the conviction of all present that the present requirements of a four-year course, with two years high school work for admission, and a seven months' term probably suits a larger number of the schools than any other scheme that could be adopted. The three-year plan with high-school diploma for admission and eight or nine months term seems the most popular concession and it would undoubtedly prevail, but it does not harmonize with the conditions which have to be met by the schools located in the South. That more time and better teaching is the demand of the profession and the public was conceded by every speaker; college men realize this more than other professional men. There is no imperative demand for more dentists at this time, but there was a tacit admission that better qualified graduates were much needed, and to secure this end some real advance in teaching time or methods must come. The statement that "better dentists were graduated twenty-five years ago than now" was not accepted as the reason for more teaching time, but that the science and art of the profession has developed to such an extent that the former teaching time and the improved methods and facilities of the present do not satisfactorily meet the demands of to-day. Just what disposition may be made of this question, which probably would never have come up had not one prominent school declined to go to a four-year course and severed its relations with the Faculty Association, can not be determined at this time. But it is evident from the discussion that an advance of some kind will be decided upon, and it will probably be practically what has already been decided upon, namely, a four-year course of seven months. It seemed to be the feeling that no sentimental reasons should be considered in taking this action, but that actual conditions should be held accountable for whatever standard hould be raised.

The meeting of the Institute of Pedagogy was one of the best, not only in attendance, but in the high and varied character of papers presented and also the discussion upon them. One could not but feel that a great change had come into this organization within the last few years. The familar faces of many of those who fostered the organization in years gone by were not present; the technical exhibits, such strong features in former meetings, were poorly represented; and even the papers presented as well as discussions have widened until all the collateral scientific subject taught seem to have been given a greater prominence than the technical subjects. It may be a question whether this is likely to accomplish as much good as under the former idea when more stress was put upon the teaching of technical subjects. It was certainly noticable at this meeting that the instructors or younger men took a less prominent part in the proceedings than the older men, or the Professors. One noticable fact was that fourteen out of thirty-two of the men put down to formally discuss the papers were absent. It does not follow, however, that the papers were not well considered, because there were present many persons who gladly availed themselves of the opportunity to talk of subjects that interest them in so peculiar a manner, and every paper received a good discussion. It is hardly possible to estimate the value of such a meeting. But from the close attention given by those present to the reading of papers and discussions one could not but feel that this organization was doing the work it was created for. Many have thought that this line of work should be done in the Faculty Association and in this way avoid holding two meetings each year when one ought to be sufficient. But any one who attended this meeting and witnessed the calm and dispassionate manner in which the Institute of Dental Pedagogics discussed the suggestion, made by Dr. G. E. Hunt in his paper on the College Curriculum, that the college course ought to be changed to a nine months' course of three years, and the nervous and excitable discussion which followed the discussion by the same men of the same proposition in the called meeting of the National Association of Faculties. In one case the bearings of the subject were taken with reference to its desirability from the pedagogic standpoint, in the other the standpoint changed to practically one of commercial practicability. It was really an interesting study in psychosis. The conclusion must be that until the National Association of Faculties ceases to have legislative functions it can not satisfactorily do the work now being so well done by the Institute of Dental Pedagogics. There were social functions of much value connected with this meeting. One was a visit to the Dental Department of the University of Buffalo. This is the school organized and so successfully conducted by the late Dr. W. C. Barrett and his associates. The college building is comparatively new and admirably adapted to the purpose for which it is used. Two sessions of the Institute were held in its well-appointed lecture room, and the members of the Institute were given a very nice luncheon at the close of one of the sessions. The absence of Dr. Barrett made the visit to the school a sad one in many respects, yet the many evidences of his great interest in educational work which were met at every turn in the building were continual reminders that his spirit was still in evidence, and his good work would certainly go on even though he can not be there in the body to direct. Dr. G. B. Snow, the new Dean, has taken up the work with his well-known devotion to the profession, and if attention to business details and honest intentions count for anything the school's future is secured already.

A trolley ride of several miles to the suburbs of the city took us to the new factory of the Buffalo Dental Manufacturing Co., a large, new three story brick building in which all the power is electrical and comes from the Niagara Falls power plant. Several hours were very profitably spent here witnessing the various processes of manufacturing the well-known dental goods of this concern. The systematic manner in which each article or part of apparatus is constructed was exceedingly interesting; very little work seems to be done by hand. A piece of brass tubing starts with a lathe which makes from one to four or five different impressions upon it, and it is then passed on through numerous lathes and hands until it finally leaves the plating room a beautifully polished vulcanizer thermometer case. And the same method carries all other parts of the vulcanizer, automatic mallet, or gas regulator to perfect completion. We haven't space or time to tell all we saw there, but the wonder is, what can they possibly do with all the things they make? The colleges will have to get busy and turn out more dentists. Perhaps one of the most delightful of the social features was the banquet given at the Iroquois Hotel Tuesday evening by the dentists of Buffalo. In was, in our estimation, the finest banquet for a large one that we ever attended. The banquet hall is a beautiful one and it was elaborately but tastefully decorated, and the banquet was served with a promptness and epicurean satisfaction that is seldom experienced. The toasts were unusually satisfying as there was little effort in the way of frivolous narrative, but each seemed happily directed to some phase of general professional interest which was in the minds of those present. It was really a first-class professional gathering. Beautiful and touching remarks were made of the absent ones who had passed away during the year. These had been so many and of such character that many deep thoughts were aroused by a brief recital of their good deeds and lives. As hosts the dentists of Buffalo and the hotel proprietors certainly know how to do the right thing in a very proper manner.

The American Society of Orthodontists began a three day's session on Thursday morning, and as a working organization this one certainly takes precedence for promptness, system and absorbing interest of the work presented. This is the first time we have had the pleasure of attending a meeting of this organization, and while we expected a profitable occasion because of an interest in the subject, and the well-known character of men, and the work accomplished by the society, we did not expect to become so much interested that we were compelled to remain until the last session. For an organization that has for its object a specialty only in the practice of such a circumscribed calling as dentistry is commonly supposed to be, this one lays tribute not only on its own profession, but all correlated arts and sciences, including sociology, biology, fine arts and medical specialties. One of the most instructive papers was contributed by an ophthalmologist in which attention was directed to the effects of abnormal development of dental organs as a cause of asymmetric development of the eyes as well as other parts of the face. There is a certain *espirit de corps* in this organization that gives it character. There is a certain loyalty and devotion to the work that accounts for the fact that it has been possible to organize such a society and to maintain such remarkable interest in its proceedings. The orthodontist seems to believe that he has a real mission to which few of the many are called; and yet to hear him talk one would imagine that the country must surely go to the bad unless more of those who are called to this work hear and accept the call. As Mr. Elbert Hubbard who gave a most interesting lecture before the society put it, the world is badly in need of the man who is "on to his job." If the orthodontists as represented in this society are not "on to their jobs," they are certainly in a fair way to get there very soon; as the evidence exhibited in the models of cases treated and the beautiful result accomplished made very clear.

The entire week was one of great pleasure and profit, and we regret that we can not reproduce it in a more satisfactory manner for our readers. The proceedings will be published in due time and good things may be culled by great diligence, but one should be present and give himself up entirely to the occasion to get the spirit and truth of such gatherings.

